# Supplementary material for: Data-Driven Technology Roadmaps to Identify Potential Technology Opportunities for Hyperuricemia Drugs
Source: Pharmaceuticals (Basel). 2022 Nov 3;15(11):1357. doi: 10.3390/ph15111357 (PMC9694917; doi:10.3390/ph15111357)
Supplement: Supplementary file 1 [file pharmaceuticals-15-01357-s001.zip › pharmaceuticals-1929159-supplementary.pdf]

Supplementary Materials: The following are available online; **Table S1.** Topic result of S components for technology layer in the time-based framework; **Table S2.** Topic result of A components for technology layer in the time-based framework; **Table S3.** Topic result of O components for technology layer in the time-based framework; **Table S4.** Topic result of S components for market layer in the time-based framework; **Table S5.** Topic result of A components for market layer in the time-based framework; **Table S6.** Topic result of O components for market layer in the time-based framework.

Table S1. Topic result of S components for technology layer in the time-based framework.

Group	Topic	Keywords
T-S-TS_1-T_1	Protein efficacy evaluation	Inflammation, antibody, chronic, microscopic, active, joint, clarity, viscosity, mucin, native…
T-S-TS_1-T_2	Modification of natural small molecule products	Extract, smilacis, rhizoma, glabrae, abcg, ointment, tacrolimus, pumpkin, narrowband, light…
T-S-TS_1-T_3	Research on the morbidity caused by taking small molecule drugs	Skin, nlrp, endometrial, colon, complication, cancer, inflammasome, blood, prostate, solid…
T-S-TS_1-T_4	Recombinant human uric acid oxidase	Urate, serum, protein, isolate, elevate, renal, fusion, recombinant, purified, oxidase…
T-S-TS_1-T_5	Drug research on xanthine oxidase inhibitors	Crystal, substitute, indazole, calcium, compound, deposition, indole, thiazolidinedione, benzotriazole, xanthine…
T-S-TS_1-T_6	Small molecule derivatives	Methoxytetrahydro, chloro, phenoxy, acetaldehyde, omethyl, oxime, benzyl, hpyran, trihydroxy, mediate…
T-S-TS_1-T_7	Study of uric acid oxidase activity	Protein, renal, organic, inhibitory, gene, fry, cross-sectional, purify, target, lectin…
T-S-TS_1-T_8	Disease-causing hyperuricemia	Syndrome, lysis, tumor, neutrophil, specie, reactive, action, oxygen, cells, increase…
T-S-TS_1-T_9	Diagnosis of hyperuricemia	Agent, edible, criterion, resonance, finding, magnetic, approach, disorder, omeract, pfkm…
T-S-TS_2-T_1	Discovery of deleterious mutation sites	Urate, vaccine, particle, metabolic, tolerogenic, layer, agent, synthetic, periodical, wiley…
T-S-TS_2-T_2	Modifying proteins to	Stress, oxidative, vascular, polymer, receiver,

	increase activity	renal, thieme, curve, uainduced, monitoring…
T-S-TS2-T3	Eutrophication causes hyperuricemia	Nonalcoholic, fatty, liver, exposure, severe, active, crystal, proteolytic, acid-base, stability…
T-S-TS2-T4	Immunogenicity analysis	Urate, serum, antibody, concentration, fragment, deposition, elevate, antioxidant, protein, inhibition…
T-S-TS2-T5	Gene mutation that inactivates the protein	Extract, gene, polymorphism, protein, renal, single, tubular, genetic, imaging, liquid…
T-S-TS2-T6	Study on combination therapy of diet and small molecule drugs	Extract, health, anti-inflammatory, direct, wine, prepare, antihyperuricemic, seed, injection, corticosteroid…
T-S-TS2-T7	Identification of amino acid residues that affect urate oxidase activity	Inflammasome, activation, gene, questionnaire, liquid, pathway, mass, efficient, spectrometry, ultraperformance…
T-S-TS2-T8	Animal model research of small molecule drugs	Compound, male, cyano, pyridine, formamide, alkoxy phenyl, kidney, receptor, derivative, mouse…
T-S-TS2-T9	Xanthine affinity chromatography	Blood, criterion, oxidase, rate, american, pressure, xanthine, college, white, mild…
T-S-TS3-T1	Affinity purification and detection of urate oxidase activity	Inflammasome, nlrp, alternative, receptor, activation, canonical, noncanonical, caspase, domain, noninvasive…
T-S-TS3-T2	Establishment of an animal model of acute arthritis	Renal, urate, serum, oxidase, xanthine, protein, oxidation, fatty, hazard, structure…
T-S-TS3-T3	Chinese medicine ingredient extraction	Extract, chinese, powder, synovitis, vascular, leaf, global, activation, mechanism, mediate…
T-S-TS3-T4	Urate anion transporter 1 (URA T1) inhibitor	Health, copolymer, college, polylactic, nsaid, acetic, derivative, acidglycolic, urateassociated, varian…
T-S-TS3-T5	Animal model construction	Urate, crystal, monosodium, nitrobenzaldehyde, synthesize, semicarbazone, dihydroxy, supplementation, mouse, dual…
T-S-TS3-T6	Diagnosis of hyperuricemia	Resonance, magnetic, blood, serum, pressure, polymorphism, multiple, reading, elevate, express…

T-S-TS_3-T_7	Etiology and complications research	Molecular, china, syndrome, strain, center, culture, microorganism, committee, bacterial, preservation…
T-S-TS_3-T_8	Constructing an animal model to analyze uric acid reabsorption	Approach, inflammation, acute, joint, pharmacological, thickness, inhibition, septum, failure, genetic…
T-S-TS_3-T_9	Small molecule efficacy research	Extract, compound, stress, oxidative, spartina, alterniflora, link, pharmacometric, pharmacoeconomic, prepare…
T-S-TS_3-T_{10}	Affinity purification and detection of urate oxidase activity	Inflammasome, nlrp, alternative, receptor, activation, canonical, noncanonical, caspase, domain, noninvasive…

Table S2. Topic result of A components for technology layer in the time-based framework.

Group	Topic	Keywords
T-A-TS_1-T_1	Small molecule modification	Alkenyl, alkyl, alkynyl, aryl, cycloalkyl, heterocyclyl, heteroaryl, reserve, monocyte, elderly…
T-A-TS_2-T_1	Eliminate/reduce urate oxidase drug immunogenicity	Reserve, objective, depression, serum, anxiety, elevados, emphasizes, emphasize, emphasise, emerge…
T-A-TS_2-T_2	Protein expression	Modification, lifestyle, yield, elicit, enable, employ, emphasizes, emphasize, emphasise, emerge…
T-A-TS_3-T_2	Small molecule modification	Hpyran, tetraol, tetrahydro, reserve, elevate, enroll, employ, equal, enzymolysis, enzyme…

Table S3. Topic result of O components for technology layer in the time-based framework.

Group	Topic	Keywords
T-O-TS_1-T_1	Pseudogene resurrection	Receptor, health, gene, cardiovascular, milk, liquid, consumption, fructose, excess, smilax…
T-O-TS_1-T_2	Anion exchange chromatography	Renal, transporter, tumor, organic, anion, multiple, lysis, syndrome, efflux, impaired…
T-O-TS_1-T_3	Strong selectivity for xanthine oxidase	Oxidase, oxidative, xanthine, activation, diagnostic, soft, mixed, code, kinase, stress…
T-O-TS_1-T_4	Align urate oxidase	Disorder, antibody, fragment, mouse,

	sequences from animals and other sources	crystal, metabolic, autosomal, dominant, crystalinduced, deposition…
T-O-TS1-T5	Small molecule modification	Diol, yloxy, phenyl, chloro, dihydroxyethyl, benzyl, tetrahydrofuran, impurity, heterocyclic, process…
T-O-TS1-T6	Combined treatment of traditional Chinese medicine and protein drugs	Urate, protein, extract, serum, renal, potential, thymus, chinese, lacking, heattreated…
T-O-TS1-T7	Rat model analysis of immunogenicity	Concentration, agent, joint, active, serum, host, clinically, molecule, meaningful, proinflammatory…
T-O-TS1-T8	Small molecule modification	Amino, ethyl, pyridyl, fluoro, pyridine, carbonitrile, isopropoxy, hpyrazol, compound, polymer…
T-O-TS1-T9	Clinical manifestations of hyperuricemia	Syndrome, enzyme, protein, severe, pain, western, metabolic, blotting, uricase, foot…
T-O-TS1-T10	Small molecule drug evaluation	Chronic, renal, action, urate, acute, uricosuric, failure, xray, kidney, neutrophil…
T-O-TS2-T1	Extend the half-life of rasburicase	Protein, liquid, compound, kinase, improve, cost, chinese, hydroxy, extract, triazol…
T-O-TS2-T2	Anion exchange chromatography	Active, methyl, transporter, pathway, anion, organic, thiazole, hydroxyphenyl, water, carboxylate…
T-O-TS2-T3	Study of protein drug activity	Oxidase, xanthine, potential, renal, inhibitor, inhibitory, agent, potent, protein, damage…
T-O-TS2-T4	Pathological manifestations of chronic hyperuricemia	Pain, chronic, progressive, inflammation, sweet, derivative, acute, kidney, damage, interaction…
T-O-TS2-T5	Preclinical studies of protein drugs	Renal, powder, endothelial, dysfunction, joint, tubular, fibrosis, proximal, oxide, release…
T-O-TS2-T6	Reduce urate oxidase immunogenicity	Urate, crystal, monosodium, oxidase, xanthine, serum, concentration, lowering, shape, birefringent…
T-O-TS2-T7	Study of probenecid side effects	Compound, renal, phenyl, amino, oxapentacyclo, hydroxyethyl, nonadeca, ethoxy, dicarbaldehyde, triene…
T-O-TS2-T8	Establishment of an animal	Health, pressure, acute, blood, potassium,

	model to evaluate the immunogenicity of human urate oxidase HR-UOX after the resurrection	extract, powder, bromo, urate, triazol…
T-O-TS$_2$-T$_9$	Small molecule drug side effects	Syndrome, metabolic, artery, thio, cyanonaphthalen, methylpropionic, pyridin, medium, culture, coronary…
T-O-TS$_2$-T$_{10}$	Animal models to evaluate the immunogenicity	Thiophen, oxane, hydroxymethyl, triol, methyl, methylphenyl, fluorophenyl, extract, serum, chinese…
T-O-TS$_3$-T$_1$	Pathological detection	Bone, china, center, preservation, water, culture, bacterial, microorganism, committee, antiinflammatory…
T-O-TS$_3$-T$_2$	Eliminate small molecule side effects	Crystal, glucose, deposit, msuinduced, wholebody, phosphate, western, realtime, blot, enzymolysis…
T-O-TS$_3$-T$_3$	Investigating the side effects of small-molecule drugs targeting the URAT1 protein	Renal, oxidase, xanthine, injury, dysfunction, chronic, molecular, joint, liver, inflammasome…
T-O-TS$_3$-T$_4$	Inducible expression of human urate oxidase	Trigger, proinflammatory, receptor, response, agent, induction, mature, acidinduced, anti, biochemical…
T-O-TS$_3$-T$_5$	Finding key amino acids	Syndrome, periodical, wiley, metabolic, interaction, target, electronic, potential, molecular, surgery…
T-O-TS$_3$-T$_6$	Protein modification	Urate, powder, transporter, serum, renal, chromatography, health, liquid, gene, genetic…
T-O-TS$_3$-T$_7$	Diet therapy with Chinese medicine	Extract, chinese, disc, solid, intervertebral, alterniflora, plantaginis, carbon, aspiration, water…
T-O-TS$_3$-T$_8$	Efficacy research of protein drugs	Consideration, mass, healthcare, epidemiology, comorbidities, pathophysiology, symptom, active, workforce, topical…
T-O-TS$_3$-T$_9$	Urate Anion Transporter 1 inhibitor and uric acid oxidase	Metabolic, mortality, cardiovascular, potential, urate, morbidity, risk, radix, elevate, light…
T-O-TS$_3$-T$_{10}$	Pathological detection	Bone, china, center, preservation, water, culture, bacterial, microorganism,

		committee, antiinflammatory…

Table S4. Topic result of S components for market layer in the time-based framework.

Group	Topic	Keywords
M-S-TS_1-T_1	The demand for traditional Chinese medicines such as angelica is increasing	Angelica, radix, xray, diffraction, figure, gentian, largeleaf, pubescens, increasingly, safety…
M-S-TS_1-T_2	Joint drug development	Chinese, fiveyear, angloswedish, growth, compound, multinational, astrazeneca, traditional, trumpet, creeper…
M-S-TS_1-T_3	Small molecule drug retail and warehouse management	Fusion, protein, oxidase, bathing, logistics, moxa, warehouse, vistaig, retail, executive…
M-S-TS_1-T_4	Market feedback of novel febuxostat febuxostat	Crystal, formic, phenyl, cyano, thiazole, isobutoxy, methyl, febuxostat, mineral, domain…
M-S-TS_1-T_5	Rasburicase has a short half-life in patients	Extract, caltrop, seed, water, cancer, indica, canakinumab, blueberry, colorectal, halflife…
M-S-TS_1-T_6	The improved half-life of porcine-baboon chimeric urate oxidase by peg modification	Advantage, secure, theta, reflection, senior, noteholders, angle, multimeric, vista, protein…
M-S-TS_1-T_7	Cooperative protein development, financing, strong alliance	Pharma, teijin, corporation, health, solid, wine, beverage, nipro, granular, reward…
M-S-TS_1-T_8	Break protectionism	Government, financial, quarter, policy, pharmaceutical, protectionist, organisation, site, nonprofit, extension…
M-S-TS_1-T_9	Development of xanthine oxidase inhibitors in Japan	Teijin, limit, cloth, driver, xanthine, oxidase, elastic, knee, growth, summary…
M-S-TS_2-T_1	Immunogenicity low drug needs	Derivative, inhibits, diazepin, triazolo, chlorophenyl, methyl, propan, compound, advantage, pharmaceutical…
M-S-TS_2-T_2	Collaborative development of small molecule drugs	Summary, pharma, teijin, healthcare, equipment, link, glucose, transporter, sodiumglucose, protein…
M-S-TS_2-T_3	Chinese herbal extracts improve biopharmaceutical	Smilacis, glabrae, absorption, chemically, block, extract, oxalate, epinephrine, generic,

	toxicity	mass…
M-S-TS2-T4	Essential amino acid residue search	Inden, dione, acetyl, pyrrolidin, methylpyrrolidin, methylbut, cyclohexane, aoctahydrospiro, ethoxy, propoxy…
M-S-TS2-T5	Asian countries increase the application of traditional Chinese medicine extracts in protein medicine	Summary, japan, tobacco, plantain, seed, asiatic, leaf, chinensis, college, fraxinus…
M-S-TS2-T6	Demand for medical devices and diet-assisted small molecule drug therapy	Summary, pharmaceutical, takeda, company, japanese, medical, device, agency, bristolmyers, kissei…
M-S-TS2-T7	Traditional Chinese medicine with small molecules to relieve drug toxicity	Chinese, compound, tetramethyl, ethoxy, triene, dicarbaldehyde, nonadeca, phenyl, himidazol, oxapentacyclo…
M-S-TS2-T8	Treatment of dampness and diuresis combined with protein drugs	Inhibitor, oxidase, xanthine, wine, chinese, sglt, herb, medicate, graveolens, ruta…
M-S-TS2-T9	Food therapy combined with small molecule drugs to alleviate drug toxicity	Extract, angelica, sinensis, powder, water, black, clematis, chinensis, officer, tea…
M-S-TS3-T1	Improve allergic reactions to pegloticase drugs	Taxus, extract, seed, chinensis, oxidase, benzbromarone, methoxy, sulfonic, antagonist, receptor…
M-S-TS3-T2	Chinese herbal extracts combined with small molecule drugs	Extract, leaf, powder, pine, chinese, cynara, scolymus, cerasus, blood, breviscapus…
M-S-TS3-T3	Combination of heat-clearing traditional Chinese medicine and small molecule medicine	Eucommia, aconiti, radix, agrestis, powder, staminate, cordyceps, bark, ulmoides, militaris…
M-S-TS3-T4	Japan-related protein drug development	Chemiphar, nippon, kurarinone, astrovirus, gmbh, verlag, goose, fortress, highlight, bivalent…
M-S-TS3-T5	Increase patient immunity	Plantain, autoimmune, diphenyl, polypeptide, antigout, syndrome, hydroxy, asiatic, active, seed…

M-S-TS3-T6	Select protein drug dosage form	Alliance, peptidream, profile, capsule, deal, entericcoated, bicarbonate, healthcare, sodium, grandis…
M-S-TS3-T7	Reduce intake of high-purine foods	Fish, smilacis, easily, molding, compression, katsuwonus, dispersible, glabrae, pelamis, peptide…
M-S-TS3-T8	Cross-company joint development	Teijin, pharma, healthcare, pharmaceutical, equipment, deal, medical, pipeline, jiangsu, summary…
M-S-TS3-T9	Combination medication	Advantage, methoxy, compound, extract, phenoxy, vinyl, fluoro, methoxyphenyl, methyl, acetic…
M-S-TS3-T10	Combination of herbs for dampness and diuresis and small molecule drugs	Advantage, inhibits, argyi, artemisiae, folium, volatile, rhizome, atractylodes, lactobacillus, wine…

Table S5. Topic result of A components for market layer in the time-based framework.

Group	Topic	Keywords
M-A-TS1-T1	Uric acid oxidase drugs	Wet, ease, eradicate, enzyme, ensures, enrich, enhances, enhance, encourage, emerge…
M-A-TS2-T1	Drug development cost estimation	Yield, emerge, expel, expect, expand, exhibit, exerts, evaluates, euryale, euonymus…
M-A-TS3-T1	Small molecule drug research	Xanthine, fuel, facilitate, extract, extend, export, expel, expands, exhibit, exerts…

Table S6. Topic result of O components for market layer in the time-based framework.

Group	Topic	Keywords
M-O-TS1-T1	Key amino acid mutations	White, sorghum, spirit, siltuximab, albumin, projection, unblinded, amino, company, liquor…
M-O-TS1-T2	Reduced side effects of small molecule drugs	Motoramyotrophic, sclerosis, spinocerebellar, lateral, ataxia, tourism, ischemic, stroke, enhance, fastdeveloping…
M-O-TS1-T3	Chinese medicine treatment	Extract, herb, drynariae, phosphopeptides, epimedium, rhizome, casein, carbonate, chondroitinsulfate, calcium…
M-O-TS1-T4	The need to develop drugs	Bind, characteristic, renal, impairment,

	with low nephrotoxicity	attractive, tighter, property, intellectual, severe, lack…
M-O-TS_1-T_5	The need for developing immunogenic drugs with low	Hydrophobic, moiety, naturally, nonnaturally, occur, positive, process, radix, lateralis, aconiti…
M-O-TS_1-T_6	Drug market risk	Active, ingredient, ministry, korean, south, oxygen, absorb, substance, property, pressure…
M-O-TS_1-T_7	Improve 'patients' quality of life	Solid, watersoluble, dispersion, carrier, diuresis, improve, multivalent, binding, enzyme, protein…
M-O-TS_2-T_1	Small molecule drug products such as zurampic and duzallo	Ethoxy, acetyl, methylbut, pyrrolidin, methylpyrrolidine, propoxy, aoctahydrospiro, dione, dimethyl, inden…
M-O-TS_2-T_2	Enterprises need to find key amino acid sites	Renal, amino, active, oxapentacyclo, phenyl, triene, nonadeca, tetramethyl, dicarbaldehyde, ethoxy…
M-O-TS_3-T_1	Reduce drug side effects	Peritonitis, hepatitis, asbestosis, radix, skin, behcet, sunburn, enteritis, silicosis, cellular…
M-O-TS_3-T_2	Market demand for drugs with low immunogenicity	Hbenzo, oxetane, carboxylic, methyl, methylbenzo, chloropyridin, imidazole, dioxotol, oxidase, piperidine…
